# ERP correlates of social conformity in a line judgment task

**DOI:** 10.1186/1471-2202-13-43

**Published:** 2012-05-03

**Authors:** Jing Chen, Yin Wu, Guangyu Tong, Xiaoming Guan, Xiaolin Zhou

**Affiliations:** 1 Center for Brain and Cognitive Sciences and Department of Psychology, Peking University, Beijing, 100871, China; 2 Key Laboratory of Child Development and Learning Science (Ministry of Education), Southeast University, Nanjing, 210096, China; 3 Department of Sociology, Peking University, Beijing, 100871, China; 4 Key Laboratory of Machine Perception (Ministry of Education), Peking University, Beijing, 100871, China

**Keywords:** Social conformity, Behavioral adjustment, Reinforcement learning, ERP, MFN

## Abstract

**Background:**

Previous research showed that individuals have a natural tendency to conform to others. This study investigated the temporal characteristics of neural processing involved in social conformity by recording participants’ brain potentials in performing a line judgment task. After making his initial choice, a participant was presented with the choices of four same-sex group members, which could be congruent or highly or moderately incongruent with the participant’s own choice. The participant was then immediately given a second opportunity to respond to the same stimulus.

**Results:**

Participants were more likely to conform to the group members by changing their initial choices when these choices were in conflict with the group’s choices, and this behavioral adjustment occurred more often as the level of incongruence increased. Electrophysiologically, group choices that were incongruent with the participant’s choice elicited more negative-going medial frontal negativity (MFN), a component associated with processing expectancy violation, than those that were congruent with the participant’s choice, and the size of this effect increased as the level of incongruence increased. Moreover, at both levels of incongruence, the MFN responses were more negative-going for incongruent trials in which participants subsequently performed behavioral adjustment than for trials in which they stuck to their initial choices. Furthermore, over individual participants, participants who were more likely to conform to others (i.e., changing their initial choices) exhibited stronger MFN effect than individuals who were more independent.

**Conclusions:**

These findings suggest that incongruence with group choices or opinions can elicit brain responses that are similar to those elicited by violation of non-social expectancy in outcome evaluation and performance monitoring, and these brain signals are utilized in the following behavioral adjustment. The present research complements recent brain imaging studies by showing the temporal characteristics of neural processing involved in social conformity and by suggesting common mechanisms for reinforcement learning in social and non-social situations.

## Background

Individuals tend to change their initial choices or opinions to match with the majority of the group they are in, a phenomenon that has been termed as social conformity [1]. Since Asch’s pioneering experiment using a line judgment task [2], different motivations underlying social conformity have been explored in a number of studies (see [3] for a review). Individuals have the desire to form an accurate interpretation of reality and when information concerning reality is insufficient, they may rely on others to provide such information or interpretation and behave accordingly (informational conformity). Individuals also have the desire to obtain approval from group members and may change their behavior to avoid social rejection, even though they privately continue to hold their original attitudes (normative conformity; see [4]). These two processes are closely interrelated and difficult to disentangle theoretically and empirically [5].

Recent studies focus on the brain structures involved in social conformity, showing that social norms (group opinions) may alter the brain activity involved in perceiving the task-relevant information. Berns et al. [6] found that erroneous responses of others could alter participants’ initial judgment in a mental rotation task and the brain activity in regions implicated in mental rotation. Zaki et al. [7] demonstrated that exposure to social norms, i.e., group opinions, affected individual’s neural representations of subjective value assigned to stimuli by increasing the activity in brain regions involved in reward processing, such as nucleus accumbens and orbitofrontal cortex (see also [8]). On the other hand, when individuals stick to their own choices in face of group members’ conflicting opinions, the brain regions involved in emotion processing, such as amygdala and caudate are activated [6]; when individuals find out that their own choices are different from the majority of the group, the brain regions associated with negative affective states, i.e., anterior insula and anterior cingulate, are activated [9], and these activations may promote the subsequent behavioral adjustment. A study by Klucharev et al. [10] found that conflict with group opinions triggered activation in the rostral cingulate zone and deactivation in the ventral striatum and signal changes in these regions predicted subsequent conforming behavioral adjustment. A follow-up study [11] demonstrated that transient down-regulation of these brain regions by theta-burst transcranial magnetic stimulation (TMS) reduced conformity, suggesting that social conformity is possibly underlined by the mechanisms that comply with principles of reinforcement learning.

**Figure 1:**
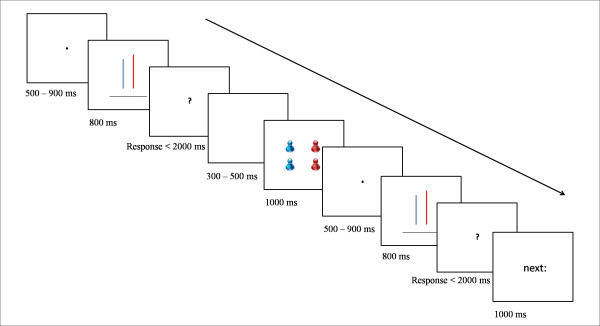
Sequence of events in a single trial.

The purpose of the present study was to complement the above neuroimaging studies by investigating the temporal characteristics of neural processing involved in social conformity. Here we developed a variant of Asch’s line judgment task in which participants were asked to judge the length of lines while their brain potentials were recorded through the event-related potential (ERP) technique. After a participant made his initial (binary) choice, he was informed of the choices of other four group members, which could be congruent or highly or moderately incongruent with his own choice. The participant was immediately given a second opportunity to respond to the same stimuli. This response would be then socially conformative (i.e., the present choice differed from the initial one but is consistent with the group’s choice) or independent (i.e., no change; see Figure 1). For the “highly incongruent” trials, a participant’s initial choice would differ from the choices of 3 or 4 (out of 4) group members; for the “moderately incongruent” trials, the participant’s initial choice would be inconsistent with the choices of 2 group members but consistent with the choices of the other two. For the “congruent” trials, the participant’s initial choice would be consistent with the choices of 3 or 4 group members.

We focused on the medial frontal negativity (MFN) or the feedback-related negativity (FRN), an ERP component that has been shown to be particularly sensitive to neurocognitive processes involved in outcome evaluation and performance monitoring. The FRN, which is a negative deflection peaking between 200 ms and 350 ms at frontocentral recording sites, has been shown to be more pronounced for negative feedback associated with unfavorable outcomes, such as incorrect responses [12], monetary losses [13], or violations of expectancy [14,15], than for positive feedback. Importantly, recent studies extended the role of FRN in outcome evaluation and performance monitoring to the social domain and found that violations of social norms, such as unfair offers in asset division, also elicit more negative-going FRN (or MFN) than fair offers [16-19]. In such studies, participants were offered either fair (e.g., 50%) or unfair (e.g., 10%) divisions of assets (monetary reward) and ERPs were time-locked to the presentation of such division schemes. Although participants were not directly provided with feedback contingent upon their actions or choices, a division scheme may be nevertheless compared with an implicit, long-established social norm concerning asset distribution and any violation of this norm by the division scheme would elicit the FRN or, more accurately, the MFN responses. Based on these studies and based on the suggestion that social group norms evoke conformity via mechanisms of reinforcement learning [10], we predicted that group choices incongruent with the participants’ own initial choices in the line judgment task would elicit more negative-going MFN responses on the participants than congruent group choices, as mismatch with others constitutes a kind of violation of social norms [3]. Moreover, the magnitude of MFN might increase as a function of the level of incongruence. Furthermore, we hypothesized that the magnitude of MFN in perceiving incongruent group choices could be differentiated according to whether the participants subsequently changed their initial choices. In other words, more negative-going MFN responses would lead to a higher likelihood of the participants subsequently changing their initial choices. Finally, across participants, the size of the MFN difference could also predict individual differences in whether changing initial choices to conform to group opinions. Such findings would provide important insights concerning the temporal characteristics of neural processes underlying social conformity.

## Results

Among the twenty-four EEG participants, four participants stated that they disbelieved the setup of the experiment in a post-test questionnaire; one participant conformed to group members in less than 5 trials for either highly or moderately incongruent conditions. These participants were excluded from further data analysis.

### Behavioral results

Trials in which the participants did not respond within time limit (2 seconds) to the initial and/or second presentation of the line stimulus were excluded from data analysis, amounting to 1.18% of the total data points (180 trials for the “highly incongruent”, 140 for the “moderately incongruent”, and 180 for the “congruent” for each participant). Trials in which the participants changed their initial choices during the second presentation of the line stimulus (i.e., exhibiting social conformity) were encoded as “change” (as opposed to “no change”) trials. We calculated the change rate as the percent of change trials out of the total trials at each level of incongruence.

**Figure 2:**
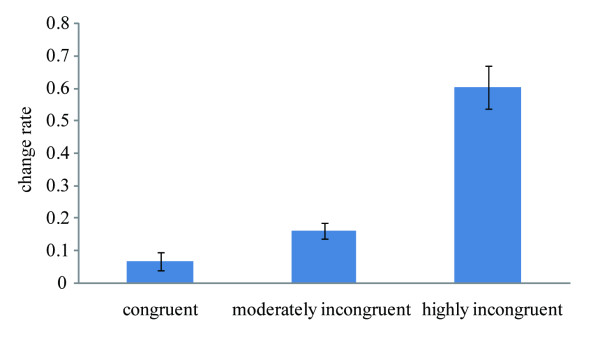
**The rate of making behavioral adjustment (i.e., making a response different from the initial one) in the second presentation of the line stimulus, depicted as a function of the incongruence level.** Error bars represented standard errors of the means.

As indicated by Figure 2, the rate of change increased as a function of the incongruence level. Analysis of variance (ANOVA) revealed a significant main effect, *F*(2, 36) = 43.81, *p* < 0.001, with the differences between conditions all being significant (*ps* < 0.01): highly incongruent (mean ± SD, 0.60 ± 0.29) vs. moderately incongruent (0.16 ± 0.11) vs. congruent (0.07 ± 0.13) condition.

### ERP results

**Figure 3:**
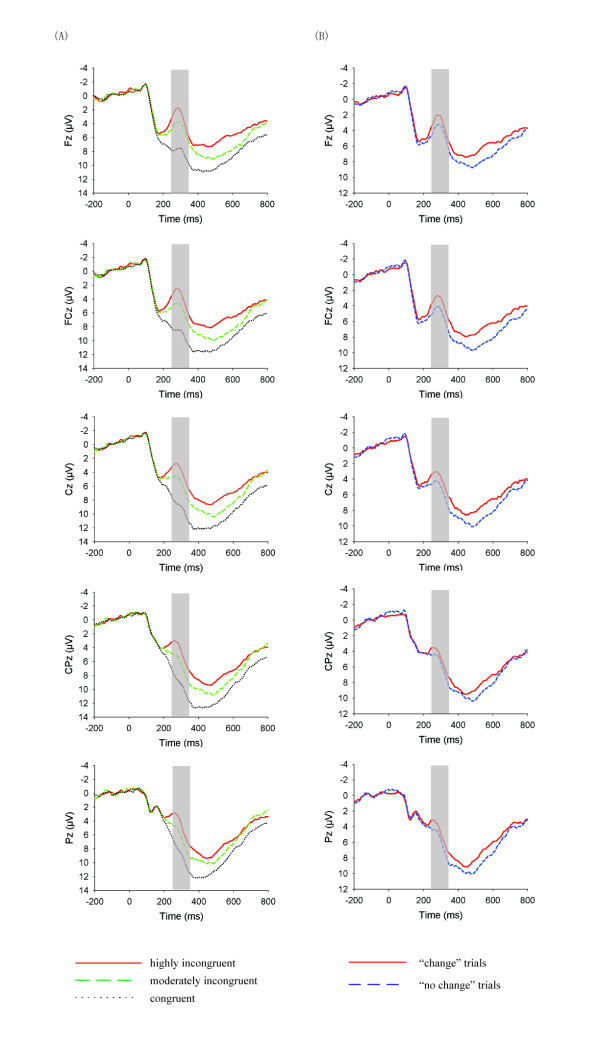
**(A) ERP responses at the midline Fz, FCz, Cz, CPz, and Pz, time-locked to the onset of the presentation of group choices and categorized by level of incongruence.** The shaded 250–350 ms window was for the calculation of the mean amplitudes of the MFN responses; **(B)** ERP responses at the midline Fz, FCz, Cz, CPz and Pz, time-locked to the onset of the presentation of incongruent group choices and categorized by subsequent behavioral tendency (change vs. no change), clasping over the highly and moderately incongruent conditions. The shaded 250–350 ms window was for the calculation of the mean amplitudes of the MFN responses.

**Figure 4:**
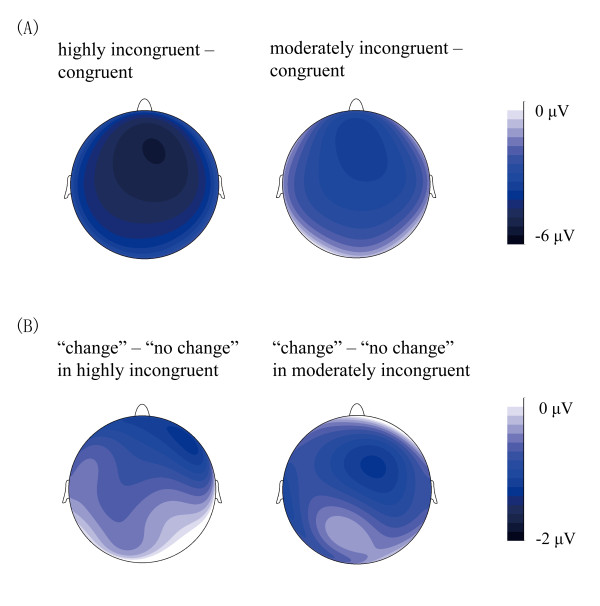
(A) topographic maps for the MFN effects evoked by group choices, categorized by level of incongruence; (B) topographic maps for the MFN differences evoked by incongruent group choices, categorized by behavioral tendency (change vs. no change).

We focused on the ERP responses time-locked to the presentation of group choices (Figure 3A), using the mean amplitudes in the 250–350 ms time window for statistical purpose. ANOVA with level of incongruence (highly incongruent vs. moderately incongruent vs. congruent), electrode row (Fz row, FCz row, Cz row, CPz row, Pz row) and laterality (left, left-middle, middle and right-middle, right) as three within-participant factors found a significant main effect of incongruence level, *F*(2, 36) = 64.57, *p* < 0.001, suggesting that the MFN responses were increasingly more negative-going for the congruent trials (8.56 ± 1.13 μV), the moderately incongruent trials (5.72 ± 1.07 μV), and the highly incongruent trials (3.98 ± 1.13 μV). The differences between conditions were all significant after Bonferroni correction, *ps* < 0.001. The main effect of electrode row was also significant, *F*(4, 72) = 5.00, *p* < 0.01, and it interacted with level of incongruence, *F*(8, 144) = 6.17, *p* < 0.001. It is clear from Figure 4A that, against the congruent condition, the congruence (i.e., the MFN) effects for both the highly incongruent and moderately incongruent conditions were larger at anterior-frontal sites.

Given that the MFN waveforms could be affected by subsequent P300 responses which are mainly associated with low frequency EEGs, we filtered the EEG data with a 2 – 20 Hz bandpass (see [14,20,21] for similar treatments). Mean amplitudes in the 250 – 350 ms time window after filtering were submitted to the 3 (highly incongruent vs. moderately incongruent vs. congruent) × 5 (Fz row, FCz row, Cz row, CPz row, Pz row) × 5 (left, left-middle, middle and right-middle, right) repeated-measures ANOVA. The pattern of effects was essentially the same as the one in the above analysis. The main effect of incongruence level was significant, *F*(2, 36) = 30.78, *p* < 0.001, indicating that the MFN responses were increasingly more negative-going for the congruent (0.26 ± 0.32 μV), the moderately incongruent (−0.54 ± 0.23 μV), and the highly incongruent (−1.05 ± 0.32 μV) trials. The interaction between electrode rows and incongruence level was also significant, *F*(8, 144) = 4.93, *p* < 0.02, suggesting that the MFN effect varied over electrode sites (the largest in the anterior-frontal region). Detailed comparisons confirmed this observation.

Note that, for simplicity of report, the following analysis on the MFN effect was restricted to the effect manifested on the anterior-frontal electrodes (F3, F1, Fz, F2, F4, FC3, FC1, FCz, FC2, FC4) where the MFN effect was the largest.

To examine whether the MFN responses in processing group choices was predictive of whether the participants would subsequently change their initial choices in the second presentation of line stimulus, we compared ERP responses for moderately and highly incongruent trials that were followed by behavioral adjustment with those trials in which the participants stick with their initial choices (Figure 3B). Here we analyzed the data in two ways. Firstly we collapsed ERP responses over the moderately and highly incongruent trials but divided them according to whether the participants changed or stuck to their initial choices in the trials (Figure 3B). ANOVA with behavioral tendency (change vs. no change) and electrode as two within-participant factors found a significant main effect of behavioral adjustment, *F*(1, 18) = 11.24, *p* < 0.01, with trials containing choice change eliciting more negative-going FRN responses (2.32 ± 1.16 μV) than trials containing no change (3.56 ± 1.12 μV). A potential problem with this analysis is that it ignored the possibility of the MFN effect for behavioral adjustment interacting with the level of incongruence. Therefore we conducted a second analysis in which the incongruent level (highly vs. moderately incongruent), behavioral tendency (change vs. no change), and electrode were treated as three within-participant factors. Given that 5 participants (out of the 19) exhibited behavioral adjustment (i.e., changing their choices) in less than 10 trials at either the high incongruent or moderately incongruent level, these participants were excluded from analysis (see [16,22] for similar treatments). ANOVA revealed a significant main effect of behavioral adjustment, *F*(1, 13) = 8.32, *p* < 0.02, with trials involving choice change eliciting more negative-going MFN responses (4.38 ± 1.40 μV) than trials involving no change (5.31 ± 1.40 μV). Importantly, this behavioral adjustment effect was not affected by the level of incongruence (on the anterior-frontal electrodes; see Figure 4B), as the interaction between behavioral tendency and level of incongruence was far from being significant, *F*(1, 13) < 1. Thus the two ways of data analysis produced the same pattern of behavioral adjustment effect.

Note, in this analysis we did not include the “congruent” condition in which the participants were shown group choices consistent with their own. Here the participant faced no conflict between their own choices and group opinions, and hence they tended in general to stick to their initial choices. For the small percentage of trials they did change their mind (7%), it was unlikely that the participants performed the change out of social conformity.

In addition, we used a trial-by-trial binary logistic regression to investigate whether the MFN response was predictive of subsequent behavior in a particular trial. Mean amplitudes in each 100 ms time window from 150 ms to 550 ms were defined as four independent variables in each trial, with the MFN referring to the second time window (250 – 350 ms, i.e., the second variable). When the four variables were entered into the regression analysis simultaneously, only the MFN predicted whether the participant would make change vs. no change choice in the highly incongruent condition, Wald = 5.76, *p* < 0.05, with the percentage of accurate prediction being 56.7%. For the moderately incongruent condition, however, this analysis did not find anything significant. Entering the MFN first into the regression and then other variables would yield essentially the same pattern of effects.

**Figure 5:**
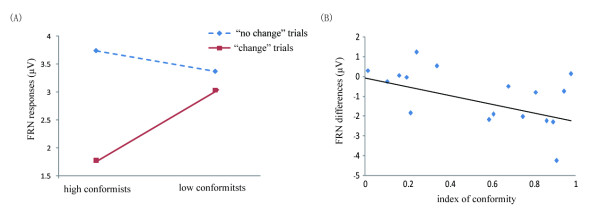
(A) mean MFN responses in the “change” and “no change” trials for the high vs. low conformist group; (B) Correlation between the individual index of conformity and the MFN difference between “change” and “no change” trials, clasping over the highly and moderately incongruent conditions.

For individual differences, we performed two types of analysis. In the first type, we split the 19 participants into two groups according to the individual index of conformity, which subtracts the rate of change in the congruent condition from the rate in the highly incongruent condition (Figure 5A). The higher the conformity index was, the more likely the participants would conform to others under social influence. After the median split, the high conformists (N = 10) had a mean index of 0.83 (SD = 0.12) while the low conformists (N = 9) had a mean index of 0.21 (SD = 0.18). ANOVA over the mean amplitudes in the MFN time window, with the participant type as a between-participant factor and behavioral tendency (change vs. no change) and electrode as two within-participant factors, revealed a significant main effect of behavioral tendency, *F*(1, 17)  = 12.81, *p* < 0.01, with the MFN responses more negative-going for the “change” (2.34 ± 1.19 μV) than for the “no change” (3.55 ± 1.36 μV) trials. Importantly, this main effect was qualified by a significant interaction between behavioral tendency and participant type, *F*(1,17) = 4.93, *p* < 0.04. Simple-effect tests showed that for the high conformist group, the MFN responses were more negative-going for the “change” trials (1.83 ± 1.64 μV) than for the “no change” trials (3.78 ± 1.59 μV), *F*(1,9) = 14.19, *p* < 0.01. However, this contrast did not reach statistical significance for the low conformist group, *F*(1, 8) = 1.22, *p* > 0.30.

In the second type of analysis, we computed the correlation, over individual participants, between the size of the MFN difference between the “change” and “no change” trials and the index of conformity (Figure 5B). This correlation was significant, *r* = −0.47, *p* < .05, indicating that the more likely a participant conformed to group members, the more negative the MFN difference was.

## Discussion

This study demonstrates that individuals are more likely to conform to group members by changing their initial choices when these choices are in conflict with the majority of group members’ choices, and this behavioral adjustment occurs more often as the level of incongruence between the individual’s and the group’s choices increases. Electrophysiologically, we found that group choices that were incongruent with the participant’s choice elicited more negative-going medial frontal negativity (MFN) or feedback-related negativity (FRN), a component associated with the processing of expectancy violation, than those that were congruent with the participant’s choice. As the level of incongruence between the participant’s choice and the group choices increased, so too did the size of the effects on the MFN or FRN. Moreover, the MFN responses were more negative-going for incongruent trials in which participants subsequently performed behavioral adjustment (“change” trials) than for trials in which they stick to their initial choices (“no change” trials). Furthermore, over individual participants, participants who were more likely to conform to others (i.e., changing their initial choices) when facing incongruent group opinions exhibited stronger MFN differences between “change” and “no change” trials than individuals who behave more independently (i.e., sticking to their initial choices).

Previous studies have demonstrated that unexpected outcomes in decision making elicit more negative-going FRN/MFN responses than expected outcomes, suggesting that the FRN/MFN is sensitive to prediction error in reinforcement learning [15,20,23,24]. Although a version of the reinforcement learning theory [25] distinguished “positive prediction error” (i.e., “the result is better than expected”) and “negative prediction error” (i.e., “the result is worse than expected”) and suggested that the two types of prediction errors are conveyed by differential impacts of the midbrain dopamine signals upon the activity in the anterior cingulated cortex (ACC), recent studies demonstrated that the MFN is possibly modulated by expectancy, with unexpected outcome eliciting more negative-going MFN responses, regardless of whether the violated expectancy is positive or negative [26]. Oliveira et al. [26] found that the FRN could be elicited by positive feedback when the participant was expecting negative feedback, and vice versa. The authors proposed an expectancy-deviation hypothesis according to which the outcome monitoring system compares the participant’s expected feedback to the actual feedback and the FRN is elicited when a mismatch between the two is detected. Wu and Zhou [15] manipulated orthogonally the reward valence, reward magnitude, and expectancy towards magnitude in a monetary gambling task. They found that the FRN effect on the feedback was sensitive not only to reward valence, but also to expectancy towards reward magnitude, with violation of expectancy eliciting more negative-going FRN responses. Thus it appears that the prediction error can be defined not only in terms of the valence of outcome but also in terms of whether the outcome fits pre-established, non-valence expectancy [15,26,27]. Further studies are needed to specifically address the differentiation between valence-based vs. expectancy-based account of the MFN/FRN effect.

Violations of social expectancy or social norms can also elicit enhanced MFN responses. It has been consistently found that unfair offers in economic exchanges evoke more negative-going MFN (or FRN) responses than fair offers [16-19]. Wu et al. went further to demonstrate that, compared with fair offers, both disadvantageous (negative) unfair offers and advantageous (positive) unfair offers elicited more negative-going MFN responses (Wu, Hu, van Dijk, Leliveld, Zhou: Brain activity in fairness consideration during asset distribution: Does the initial ownership play a role?, submitted).The MFN effect may reflect the detection of social expectancy violation as egalitarian distribution of assets is an expected social norm [28,29]. During evolution, the human brain may have developed specific mechanisms to detect ongoing deviations from social norms [30]. It is possible that these mechanisms share the same neural correlates as those engaged in predicting errors during non-social reinforcement learning [10,31]. The MFN can therefore reflect not only the encoding of prediction errors for monetary reward or performance feedback but also violations of expectancy toward social norms. In the present study, individuals could compare their initial choices with opinions of other group members and the difference with others could be encoded as a prediction error. A recent ERP study on social conformity also suggested that social deviance activates the brain’s error-monitoring system [32].

In this study, we also found that MFN responses in processing group choices incongruent with the participants’ own choices can be predictive of whether they would subsequently change their mind when they were given a second opportunity to make line judgment (c.f., [22]). At both levels of incongruence, trials in which the participants changed their mind showed more negative-going MFN responses than trials in which they stick to their initial judgment. The reinforcement-learning theory of MFN [25,33] suggests that the MFN reflects the coding of prediction error in the midbrain dopamine system, which sends signals to the anterior cingulate cortex (ACC) and guides action selection mediated by the ACC through the reinforcement of action associated with positive reward and the punishment of action associated with negative outcomes. Social conformity can be considered a type of goal-directed action in which the goals of behavior include maximizing the rewards following accurate performance and social acceptance, and minimizing the punishment following erroneous response and social rejection [3]. In the present study, the more negative going MFN responses for the “change” trials, as opposed to the “no change” trials, demonstrated stronger neural signals sent to ACC, which guided subsequent behavioral adjustment (i.e., actions consistent with group opinions or social norm). Indeed, a recent fMRI study also showed that the amplitude of conflict-related signal in brain regions implicated in reinforcement learning, i.e., rostral cingulate zone and the ventral striatum, can predict subsequent behavioral conformity [10].

The account that social conformity is instantiated via reinforcement learning mechanisms is further strengthened by the finding that individuals who were more likely to conform to the others exhibited a stronger MFN difference between “change” and “no change” trials when compared with individual who were less likely to change their mind. Previous studies have shown that the MFN responses are sensitive to individual differences along different dimensions, including personality or morality. For example, Yeung et al. [34] reported a correlation between the MFN amplitude and the participants’ rating on how much they felt to be involved in the gambling task, with larger MFN amplitudes corresponding to higher involvement ratings. Boksem and De Cremer [16] found that the MFN amplitude was more pronounced in perceiving unfair, as opposed to fair, offers and this effect was larger for participants with higher concerns for fairness than for participants with lower concerns. Violation of social norms is a kind of prediction error that can be utilized as reinforcement learning signal for subsequent behavioral adjustment. The more significant the prediction error is valued by an individual, the more likely he would subsequently change mind to conform to others (see also [10]).

Note that, in the above discussion, we have largely categorized the conformity effect we observed as “normative conformity” and attributed the desire to be consistent with others in choice selection as a kind of social reinforcement. However, it is also possible that participants had simply used others’ choices in line judgment as a source of information in order to make more accurate judgment (i.e., informational conformity). As most studies on social conformity, the experimental design we used could not allow us to definitely differentiate the two types of conformity. A possible way to improve the design is to include a control condition in which the group opinions come from computer programs (see [35]). However, if the computer programs generate choices based on stored knowledge, participants might anthropomorphize the computer programs (i.e., treating the computers as humanized agents), and the conformity effect obtained in this situation can still be taken as being out of normative conformity; if the computer programs generate choices randomly, participants might treat these choices differently. Indeed, providing participants with "buy" or "not buy" choices of stocks randomly produced by four chimpanzees [36] or providing participants with attractiveness judgment of human faces randomly produced by computers [11] did affect participants’ choice behavior, but these effects were much weaker than the impacts of group choices produced by human peers.

Moreover, group opinions produced by human peers and group choices generated by computer programs elicit differential neural signals in brain regions implicated in reinforcement learning but not in brain regions involved in sensory-perceptual processing [11]. Taken together, one might conclude that the conformity effect observed in this and some other studies is essentially out of normative conformity. That is, participants’ subsequent behavioral adjustment “is mediated by the reinforcement learning mechanism in which both reward for being aligned with group and aversion to being non-aligned may have acted as reinforcers” [11].

Another issue that needs discussion concerns whether the MFN effect observed might be explained in terms of attention devoted to the congruence of group opinions. In particular, the participants might have paid less attention at the start of some trials, making them ignore the group opinions. Consequently they showed smaller neural responses to incongruent group opinions and a weaker tendency to subsequently adjust their choices. However, this line of argument seems implausible as the P300, which is generally believed to reflect the distribution of attentional resources [37], was actually more positive for the “no change” trials than for the “change” trials (Figure 3B).

## Conclusions

By manipulating the level of (in)congruence between the participants’ initial choices and group members’ choices in a line judgment task, the present study demonstrated that 1) incongruent group choices would elicit more negative going MFN responses than congruent ones when the participants were presented with the choices; 2) incongruent group choices in trials in which the participants changed their mind when given the second opportunity to make line judgment elicited more negative-going MFN responses than incongruent group choices in trials in which the participant stuck to their original opinion; 3) over individual participants, participants who were more likely to conform to others exhibited stronger MFN differences between “change” and “no change” trials than those who were not. These findings suggest that incongruence with group choices or opinions (which acts as a kind of social norm) can elicit brain responses that are similar to those elicited by violation of non-social expectancy in outcome evaluation and performance monitoring, and these brain signals can be utilized in the following behavioral adjustment. The present study complements recent brain imaging studies by showing that the brain rapidly computes the social norm based on group members’ opinions and compares one’s own action with the norm. The study also suggests common mechanisms for reinforcement learning in social and non-social situations.

## Methods

### Participants

Twenty-four undergraduate and graduate students (13 females; mean age 22.5 years, SD = 1.93) participated in the experiment. Four students, who were strangers to the participants, were recruited as confederates. To exclude possible influence of sex on social conformity, each EEG participant was grouped with 4 same-sex confederates [38].

All the participants were right-handed and had normal or corrected-to-normal vision. They had no history of neurological or psychiatric disorders. Informed consent was obtained from each participant before the test. The experiment was performed in accordance with the Declaration of Helsinki and was approved by the Ethics Committee of the Department of Psychology, Peking University. Each participant was paid 60 Chinese yuan (about USD$ 9.5) as basic payment and was informed that additional monetary reward would be paid according to their performance in the task.

### Design and procedures

The experiment used a one-factor within-participant design with three levels of group choice. For the highly incongruent condition, three or four group members made choices different from the participant’ initial choice; for the moderately incongruent condition, two group members made choices different from the participant’s while the other two members made the same choices; for the congruent condition, one or no group member made choices different from the participant’s.

When a participant came to the laboratory, he and the four confederates were told that they would sit in separate rooms to complete a task together through the computer network. By assigning the participant and the confederates pre-determined cards, they were ostensively led to separate cubicles to play different roles in the task. The participant was then told that he as well as the other four group members would finish a line judgment task together. He was also informed of the procedures of the experiment (Figure 1). That is, at the beginning of each trial, the participant was presented with two parallel vertical lines, with a length of either 5.5 or 6.0 cm, on either left or right side of the screen (with one color appearing at one side in half of the trials) and a horizontal black line (with a length of 6.0 cm). He had to judge which one of the two vertical lines is of the same length as the horizontal one by pressing a button with the index finger of the left or right hand (i.e., a binary judgment). The position of the horizontal line was either on the top of or on the bottom of the two vertical lines while the relative positions of the two vertical lines varied slightly along the vertical orientation over trials. Participants reported in a post-experiment questionnaire that it was almost impossible for them to be sure which vertical line (with a difference of 0.29 degree in visual angle between the lines) was of the same length as the horizontal line. A detailed examination of the participants’ responses showed that the accuracy of the participants’ responses (i.e., choosing the vertical line with 6.0 cm) was 43.38%, which did not differ significantly from the chance level (50%), *t*(18) = 1.27, *p* > 0.1.

The participant was then presented with a frame indicating, through coloring cartoon figures, how many of the 4 other group members had chosen the red or blue lines. The group choices were predetermined by a computer program without the participant’s knowledge, and red or blue lines were randomly assigned. The participant was shown the same line stimulus again, and was instructed to indicate his choice the second time by pressing a response button. The participant was informed before the experiment that the computer would record his responses and the extra payment was dependent upon the accuracy of his second choice in each trial. The time line of the presentation of each frame in each trial was illustrated in Figure 1.

The participant was comfortably seated about 1.0 m in front of a computer screen in a dimly lit room. The experiment was administered on a computer with a Del 22-inch CRT display using Presentation software (Neurobehavioral System Inc.) to control the presentation and timing of the stimuli. For the highly incongruent condition, all the four group members’ choices were different from the participant’s in 120 trials and three members’ choices were different in 60 trials. For the moderately incongruent condition, two group members’ choices were different from the participant’s in 140 trials. For the congruent condition, three group members (but one) had the same choice as the participant in 60 trials, and all the four group members had the same choice as the participant in 120 trials. The 500 trials were randomly mixed and were divided in equal numbers into 5 test blocks with the restriction that no more than three consecutive trials were at the same incongruence level. A practice block of 30 trials in which the participants underwent the same procedure as that in the formal test was administered to familiarize the participants with the experiment. Participants were debriefed, paid, and thanked at the end of the experiment.

### EEG recording and analysis

EEGs were recorded from 64 scalp sites using tin electrodes mounted in an elastic cap (Brain Products, Munich, Germany) according to the international 10–20 system. The vertical electrooculogram (VEOGs) was recorded supra-orbitally from the right eye. The horizontal EOG (HEOG) was recorded from electrodes placed at the outer canthus of the left eye. All EEGs and EOGs were referenced online to an external electrode, which was placed on the tip of nose, and were re-referenced offline to the mean of the left and right mastoids. Electrode impedance was kept below 10 kΩ for EOG channels and below 5 kΩ for all other electrodes. The bio-signals were amplified with a bandpass from 0.016 to 100 Hz and digitized on-line with a sampling frequency of 500 Hz.

Separate EEG epochs of 1000 ms (with a 200-ms pre-stimulus baseline) were extracted offline, time-locked to the onset of group opinion. Ocular artifacts were corrected with an eye-movement correction algorithm that employs a regression analysis in combination with artifact averaging [39]. Epochs were baseline-corrected by subtracting from each sample the average activity of that channel during the baseline period. All the trials in which EEG voltages exceeded a threshold of ± 80 μV during recording were excluded from further analysis. The EEG data were low-pass filtered below 30 Hz.

For the MFN, the mean amplitudes in the time window of 250–350 ms were analyzed. This time window was selected according to the classical definitions for the MFN and according to visual inspection of waveforms. The Greenhouse-Geisser correction for violation of the assumption of sphericity was applied where appropriate. The Bonferroni correction was used for multiple comparisons.

The mean number of trials that was entered in MFN analysis was 132.2 (ranging from 79 to 175) per participant for the highly incongruent condition, 100.1 (from 52 to 131) for the moderately incongruent condition, and 133.7 (from 71 to 173) for the congruent condition. After discarding the five participants who had less than 10 “change” trials in either the highly or the moderately incongruent condition, for the remaining 14 participants, the mean number of trials that was entered into the “change” vs. “no change” comparison was 70.4 (for “change”, ranging from 27 to 156) and 54.9 (for “no change”, ranging from 17 to 111) per participant in the highly incongruent condition and were 23.1 (ranging from 11 to 38) and 73.3 (ranging from 12 to 106) per participant in the moderately incongruent condition.

It is clear from Figure 3 that the choice congruence effect and difference between “change” and “no” change” trials appeared not only in the MFN window, but also in a later, possibly the P300, time window. But given that the pattern of effects in the later time window was essentially the same as the one for the MFN, we did not report the analysis of the effects in this window.

## Misc

Jing Chen, Yin Wu contributed equally to the work

## Authors’ contributions

JC, YW, GT, XG and XZ codesigned the experiment. JC and GT performed the experiment and the data analysis. JC, YW and XZ wrote the paper. All authors read and approved the final manuscript.
